# Object-centric diffusion policies for real-world robotic-arm imitation learning

**DOI:** 10.3389/frobt.2026.1829436

**Published:** 2026-07-15

**Authors:** Prashant Reddy Kasu, Dugan Um

**Affiliations:** 1 College of Computer Science and Engineering, Computer Science, Texas A&M University - Corpus Christi, Corpus Christi, TX, United States; 2 College of Computer Science and Engineering, Mechanical Engineering, Texas A&M University - Corpus Christi, Corpus Christi, TX, United States

**Keywords:** diffusion policy, DINO (DETR-based object detection), imitation learning, multimodal action generation, real-world robotics, robotic manipulation, transformer-based vision

## Abstract

Imitation learning in complex, unstructured environments remains challenging due to the difficulty of grounding perception in physically meaningful representations and the need to model multimodal action distributions. Existing approaches often rely on unstructured pixel-level feature encodings or stochastic latent-variable decoders, which can lead to brittle attention in cluttered scenes. In this work, we present a novel integration of detector-based visual representations with conditional diffusion modeling (DINO + CDP) for real-world robotic imitation learning. Our framework utilizes a DINO object detection transformer to extract spatially-grounded object-query embeddings that serve as the conditioning signal for a diffusion-based policy. A primary contribution of this work is the systematic quantification of how scene complexity—measured via image entropy—affects robotic policy performance. By comparing rigid-object baselines with complex biological plant scenes, we demonstrate that organic morphology induces a measurable increase in pixel-level uncertainty that degrades standard pixel-centric models. Our results show that DINO + CDP mitigates this degradation by grounding action generation in stable object-level features. We evaluate our approach using a fully real-world manipulation dataset collected without simulation or synthetic pre-training. To isolate the impact of our architectural choices, we conduct a comparative study within a unified framework against convolutional (CNN-MLP), transformer-patch (ViT), and latent-variable (DETR + CVAE) variants. Experimental results in a robotic-arm biocell setup demonstrate that object-query-conditioned diffusion significantly improves task success rates, produces smoother trajectories, and exhibits superior robustness to high-entropy visual inputs, establishing a scalable pathway for imitation learning in challenging agricultural domains.

## 1 Introduction

Robotic manipulation in real-world environments requires precise interaction, adaptability to uncertainty, and reliable perception-to-action mapping. Tasks involving deformable objects, occlusions, and partial observability remain particularly challenging, as they demand both accurate scene understanding and the ability to generate consistent control actions under noisy sensory inputs. Imitation learning has emerged as an effective paradigm for acquiring such skills directly from demonstrations. However, its performance in real-world settings is often limited by ambiguities in visual perception and the presence of multimodal action distributions.

Traditional visuomotor imitation learning approaches typically rely on convolutional neural networks (CNNs) combined with multilayer perceptrons (MLPs) to map raw pixel observations to control commands. While computationally efficient, these methods depend on globally aggregated features and may lack sufficient spatial structure for tasks requiring precise localization. Vision Transformer (ViT)-based models improve representational capacity by capturing long-range dependencies through self-attention over image patches.

However, patch-based representations do not inherently produce entity-aligned or object-centric tokens unless paired with appropriate supervision signals or architectural inductive biases. As a result, while ViTs can effectively encode spatial information, additional mechanisms are often required to explicitly associate features with physically meaningful entities in the scene.

To introduce explicit object- and region-centric representations, we explore the use of structured visual outputs derived from transformer-based detection models. In particular, architectures such as denoising DETR employ learned queries that attend to image features and produce a fixed set of region-level embeddings associated with salient parts of the scene. These query-based representations provide a structured interface between perception and control, enabling downstream policies to operate on spatially meaningful features rather than unstructured global embeddings.

In parallel, action generation in imitation learning has evolved from deterministic regression to probabilistic generative modeling. Conditional variational autoencoders (CVAEs) model multimodal action distributions through latent variables, but often suffer from issues such as mode averaging and instability in long-horizon predictions. Diffusion-based policies offer an alternative by generating actions through iterative refinement, allowing more stable modeling of complex and multimodal behaviors.

Diffusion-based generative models have recently emerged as a powerful alternative for modeling complex, high-dimensional distributions. Originally developed for image synthesis, diffusion models learn to reverse a gradual noise corruption process through iterative denoising. When applied to control, diffusion policies generate action sequences by progressively refining noisy trajectories conditioned on sensory input. This iterative refinement mechanism provides improved stability and expressiveness compared to single-pass decoders, enabling more faithful modeling of multimodal behaviors.

Despite the independent progress in object-query-based perception and diffusion-based policy learning, limited work has investigated their joint integration in real-world agricultural manipulation tasks. Many existing studies focus on simulation environments or single-arm platforms, which may not fully capture hardware-level synchronization challenges, environmental variability, and sensing noise. Real-world validation is particularly important in biological interaction scenarios, where deformability, occlusion, and environmental perturbations introduce significant complexity.

In this work, we present a diffusion policy framework for real-world robotic imitation learning. Our approach integrates structured visual perception with iterative action generation to address both representation and multimodality challenges. Multi-view observations are processed using a DINO object detection transformer (DETR with Improved DeNoising Anchor Boxes), which extracts object-query-based visual embeddings and spatial representations from the scene. We note that, in this work, “DINO” refers specifically to the DETR-based object detection framework and not the self-supervised DINO representation learning model. These structured embeddings are fused with robot proprioceptive state information and provided as conditioning inputs to a conditional diffusion policy. The diffusion model generates consistent and stable robotic action trajectories through iterative denoising, directly modeling multimodal behavior without relying on latent-variable bottlenecks.

**FIGURE 1:**
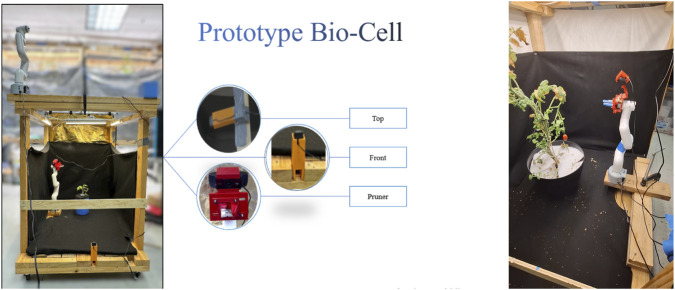
Biocell prototype and tomato plant used for demonstration collection and model evaluation.

We validate the proposed framework on a real-world biocell manipulation setup using an Actor–Imitator system, where demonstrations are collected via a teleoperated actor arm that provides supervisory trajectories (Figure 1). During evaluation, only the imitator arm executes the learned policy. All models are trained and evaluated exclusively on physical hardware without simulation pretraining. We compare our approach against CNN-MLP policies, Vision Transformer-based policies, and a DETR combined with a CVAE baseline under identical experimental conditions. Quantitative and qualitative evaluations demonstrate improved task success rates, smoother trajectory execution, and enhanced execution stability.

While the experimental evaluation is conducted in an agricultural setting, the primary focus of this work is on the integration of structured visual representations with diffusion-based control policies for real-world robotic imitation learning. Although prior work has explored transformer-based visual encoders and diffusion-based policies independently, their integration remains underexplored, particularly in real-world robotic systems. Existing diffusion policies typically rely on pixel-level or patch-based visual embeddings, which lack explicit alignment with semantically meaningful scene entities. In contrast, our approach introduces object-query-conditioned diffusion, where structured detection tokens derived from a transformer-based detector provide spatially grounded representations of physical entities. Compared to latent-variable approaches such as CVAEs, which rely on stochastic bottlenecks and can suffer from mode averaging, the proposed framework directly models multimodal action distributions through iterative refinement. This combination enables more stable long-horizon action generation while preserving interpretable and spatially meaningful perception, distinguishing our approach from prior pixel-centric and latent-variable methods. The contributions of this work are summarized as follows:

- We introduce a real-world imitation learning framework that integrates transformer-based detection representations with conditional diffusion policies that integrates DINO-based perception with conditional diffusion policies for manipulation.
- We present a hardware-validated biocell manipulation benchmark using coordinated Actor-Imitator robots operating on a biological interaction task.
- We provide systematic empirical comparisons against convolutional, transformer-based, and latent-variable baselines under consistent real-world conditions.
- We demonstrate that combining structured object representations with diffusion-based action modeling improves multimodal coordination and execution stability in real-world systems.

These findings suggest that object-query-based diffusion policies provide a robust and scalable pathway toward advancing real-world robotic arm imitation learning.

## 2 Related work

### 2.1 Agricultural robotics

Imitation learning has emerged as a key framework for mapping visual perceptions directly into executable robotic actions from human demonstrations. Early foundational benchmarks established direct behavior cloning pipelines across diverse visual spaces (Pomerleau, 1988). However, continuous execution streams often face performance bottlenecks due to compounding spatial drift and error propagation. To address these out-of-distribution challenges, robust data collection frameworks introduce synthetic noise paths to systematically correct policy behavior when experiencing structural deviations (Ross et al., 2010; Laskey et al., 2017). Furthermore, recent work mitigates training uncertainties by integrating bayesian ensemble approximations to monitor operational tracking variances (Menda et al., 2018) while isolating the precise dataset constraints that impact localized physical manipulations (Mandlekar et al., 2021).

To better capture continuous action paths without averaging discontinuous trajectories, recent trends favor structural implicit and generative architectures. Energy-based modeling paradigms parameterize continuous manipulation choices implicitly, avoiding the drawbacks of deterministic action outputs (Yilun Du and Igor Mordatch, 2019; Florence et al., 2021). Similarly, deep score-based generative structures and conditional diffusion models have set new benchmarks by framing path generation as a stochastic denoising sequence (Song et al., 2021), preserving the unique geometric features found in human trials (Chi et al., 2023). Beyond trajectory modeling, tokenized attention backends have scaled parameter capacities dramatically (Vaswani et al., 2017), leading to large-scale multimodal foundations such as the Robotics Transformer (RT-1) that excel at zero-shot task execution across physical boundaries (Brohan et al., 2022).

To protect high-capacity sequence models from tracking irrelevant pixel variations in cluttered scenes, modern architectures focus heavily on scene grounding through spatial and object-centric tokens. End-to-end transformer detectors generate highly reliable object embeddings that provide durable semantic and physical continuity across frames (Nicolas et al., 2020). When integrated with unified multi-task networks, these object-level tokens allow systems to bind linguistic commands to explicit per-pixel affordance fields (Shridhar et al., 2021) or navigate multi-layered 3D spatial configurations using voxelized action spaces (Shridhar et al., 2022). Moreover, synthesizing novel perspectives via neural radiance fields offers an alternate way to enrich datasets, shielding policies from unexpected camera displacements by creating photorealistic view updates from sparse sensor feeds (Zhou et al., 2023).

Deploying these vision-action models successfully depends on the reliability and precision of the tracking frameworks used during human demonstrations. Recent advancements replace complex tracking apparel with markerless, vision-based capture systems that map human hand geometries directly to high-DOF dexterous setups in real-time (Jeffrey et al., 2020). When augmented with mixed-reality interfaces, these configurations streamline real-world data collection through immersive visual streaming loops (Pandian Arunachalam et al., 2022). This structural tracking fidelity has proven essential for learning intricate material behaviors, such as the dynamic, high-speed configurations needed to unfold non-rigid cloth (Ha and Song, 2021). Finally, dense tracking layers facilitate the extraction of self-supervised spatial correspondences across environments to align features without hand-labeled data (Peter et al., 2019), serving as an effective control baseline for managing synchronized multi-arm assemblies and complex dual-arm interactions (Smith et al., 2012).

A strategic response to ongoing production shortages and the need for sustainable farming practices is the integration of robotics into agriculture, as highlighted in recent comprehensive reviews of agricultural automation (Rajput and Jain, 2021; Gonzalez et al., 2021). Emerging agricultural robotic systems demonstrate significant potential to enhance productivity while promoting responsible resource use and environmental stewardship (Arguello et al., 2020; Das and Shokoohi, 2021). These systems are applied across a wide range of tasks, including land preparation, sowing, crop monitoring, treatment, and harvesting (Rajput and Jain, 2021; Frison et al., 2021). However, despite substantial technological progress, current robotic platforms continue to face challenges related to sensor integration, perception robustness, mobility in unstructured terrain, and adaptation to diverse agricultural environments (Tian et al., 2021; Gonzalez et al., 2021). Many existing solutions are task-specific, limiting their flexibility and broader applicability in dynamic farming contexts (Henke et al., 2021; Martinez et al., 2022). Advancements in computer vision, machine learning, communication technologies, and control architectures are therefore essential to improve system robustness and autonomy (Arguello et al., 2020; Miller and Smith, 2021; Smith et al., 2020).

At the same time, global population growth—projected to reach 9.8 billion by 2050—places increasing pressure on farmers to boost food production, intensifying the need for automation technologies that can mitigate labor shortages and enhance efficiency (Joshi, 2021; Zhang Y. et al., 2021). Robotics plays a critical role in this transition by automating repetitive and labor-intensive tasks while enabling data-driven farm management through advanced sensing and algorithmic decision-making (Das and Shokoohi, 2021; Arguello et al., 2020; Das et al., 2021). Nevertheless, full automation of complex operations such as harvesting remains particularly challenging in unstructured environments where adaptability and dexterous manipulation are required (Bello et al., 2020; Henke et al., 2021).

**FIGURE 2:**
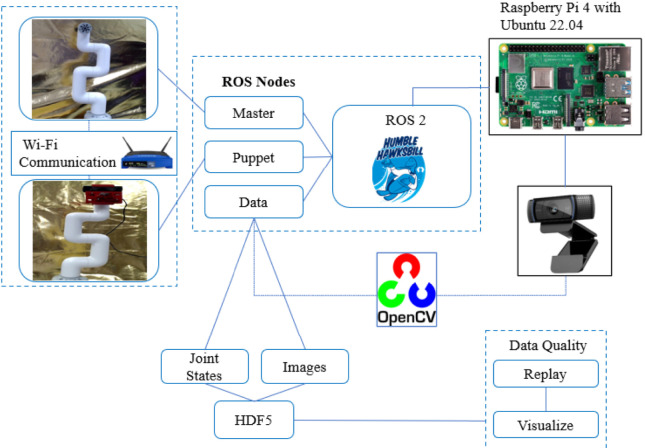
System architecture overview of the actor-imitator robotic biocell platform, including actor–imitator roles, sensor integration, multi-camera perception, ROS 2 communication, and data logging pipeline.

To address these challenges, this research proposes a actor-imitator robotic system comprising an Actor robot controlled by a human operator and an Imitator robot that replicates the demonstrated actions in real time. Teleoperation-based agricultural robotics has been explored as a practical bridge toward autonomy, particularly for precision farming and harvesting tasks (Lopes et al., 2022; Rodrigues et al., 2021; Zhang X. et al., 2021). By combining human expertise with robotic replication, the proposed framework aims to enhance operational efficiency while progressively enabling higher levels of automation. Furthermore, emerging paradigms such as swarm robotics—where multiple robots collaborate to improve coverage, precision, and productivity—demonstrate promising future directions for scalable autonomous agricultural systems (Meireles and Santos, 2021; Noor et al., 2022). Despite substantial advancements, the literature continues to emphasize the need for adaptable, autonomous, and cooperative robotic systems capable of addressing labor shortages and improving productivity across varied agricultural scenarios (Gonzalez et al., 2021; Martinez et al., 2022). The actor-imitator robotic architecture presented in this work contributes to this objective by advancing automation strategies for agricultural manipulation tasks and supporting the evolution toward more flexible and scalable robotic farming solutions (Figure 2).

Fine manipulation in agricultural robotics has been widely studied in harvesting and pruning tasks, where robots must perceive partially occluded biological structures, localize task-relevant regions, and execute precise contact-rich actions. Prior systems have demonstrated important progress in grapevine pruning, pepper harvesting, apple harvesting, and tomato manipulation. However, many existing approaches rely on modular perception-control pipelines, task-specific end-effectors, geometric planning, or deterministic control policies. In contrast, the proposed DINO + CDP framework combines detector-based object-query representations with conditional diffusion-based action generation, enabling the policy to model multimodal manipulation trajectories while maintaining object-centered visual grounding in cluttered biological scenes. Table 1 represents the comparison fine-manipulation robotic systems in agricultural applications.

**TABLE 1:** Comparison of representative fine-manipulation robotic systems in agricultural applications and positioning of the proposed framework.

Study	Application	Perception/Control strategy	Main contribution	Limitation compared with proposed DINO + CDP
Botterill et al.	Grapevine pruning	3D vine reconstruction and robotic pruning-point selection	Demonstrated autonomous robotic grapevine pruning	Relies on geometry-driven pruning decisions without learned multimodal action generation
Arad et al. (SWEEPER)	Sweet pepper harvesting	RGB-D perception with robotic harvesting manipulator	Developed an autonomous greenhouse sweet-pepper harvesting robot	Primarily task-specific harvesting pipeline without imitation-learning-based visuomotor policy generation
Kang et al.	Apple harvesting	Deep learning fruit detection and grasp estimation	Proposed real-time fruit localization and grasp estimation	Focuses mainly on target recognition and grasping rather than object-conditioned action generation
Jun et al.	Tomato harvesting	3D perception and manipulator planning	Integrated tomato localization and robotic harvesting control	Uses modular perception-planning-control pipeline instead of end-to-end object-centric policy learning
Zhang et al.	Apple harvesting	Laser-camera scanning and robotic picking system	Improved clustered-fruit recognition for robotic harvesting	Does not model multimodal manipulation trajectories using generative policies
Ma et al.	Tomato branch pruning	Structural optimization of robotic pruning arm	Optimized manipulator structure for pruning operations	Focuses primarily on manipulator mechanics rather than learned visuomotor control
Ning et al.	Sweet pepper harvesting	YOLO-based detection with picking-order planning	Improved target recognition and picking sequence optimization	Limited emphasis on closed-loop generative manipulation policies
Mahmoudi et al.	Agricultural imitation learning survey	Review of imitation-learning methods in agricultural robotics	Summarized imitation-learning trends in agricultural robotics	Survey contribution without proposing detector-conditioned diffusion policies
Proposed DINO + CDP	Real-world robotic-arm imitation learning	Object-query-conditioned diffusion policy with detector-based visual grounding	Integrates object-centric perception and multimodal diffusion-based action generation for robust manipulation in cluttered biological scenes	Demonstrates improved robustness to occlusion, clutter, and high-entropy visual conditions relative to pixel-centric and latent-variable baselines

### 2.2 Generative models for visuomotor control

The evolution of imitation learning has seen a shift from deterministic regression to generative modeling to handle the multimodality of human demonstrations. Early approaches utilized latent-variable models such as Conditional Variational Autoencoders (CVAEs) (Kingma and Welling, 2013), which capture distribution modes but often suffer from training instability. This led to the development of Energy-Based Models (EBMs) (Yilun Du and Igor Mordatch, 2019), which represent the action distribution through an implicit energy function and utilize iterative Langevin dynamics for sampling. While EBMs offer high expressivity, they are often difficult to train and computationally expensive. More recently, Diffusion Policies (Song et al., 2021) have emerged as a robust alternative, adopting a similar iterative refinement process but with significantly improved sampling efficiency and training stability. By framing action generation as a denoising process, diffusion models retain the multimodal advantages of EBMs while providing more consistent performance in complex manipulation tasks.

## 3 Materials and methods

### 3.1 Overview of the system and study design

**FIGURE 3:**
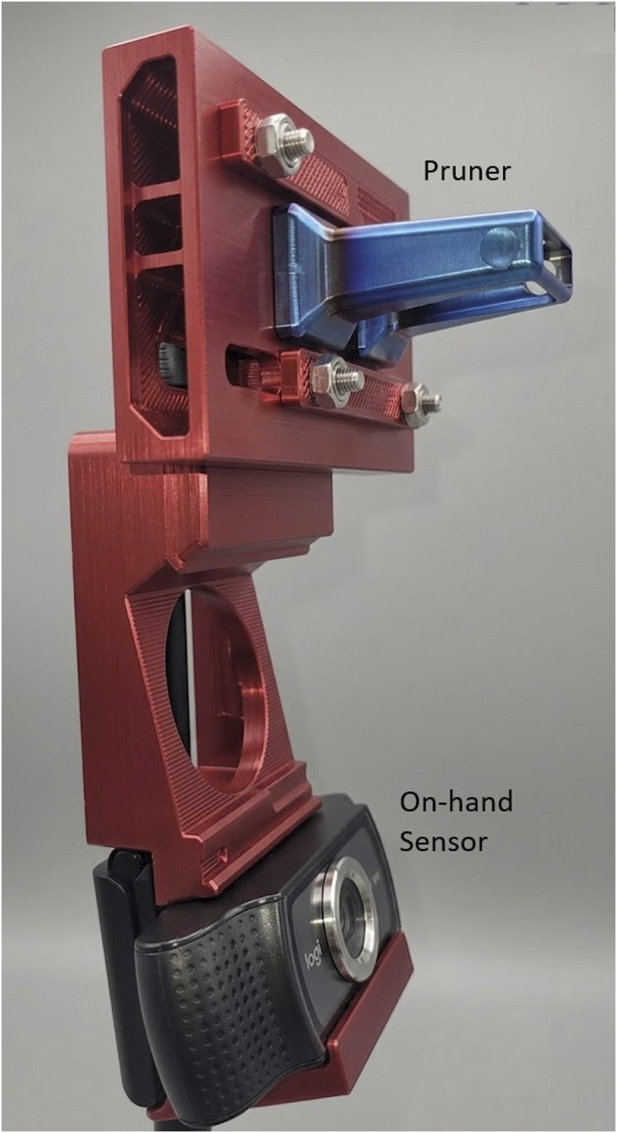
Custom pruner end-effector design. A pinion-and-rack mechanism converts rotational motion into linear actuation for cutting operations.

We developed and evaluated a real-world actor-imitator robotic platform for indoor agricultural manipulation in a controlled biocell environment. The system consists of two six-degree-of-freedom (6-DOF) MyCobot robotic manipulators operating in a shared workspace containing a tomato plant (Figure 3). The platform is designed for teleoperated demonstration collection (actor–imitator configuration) and for subsequent imitation-learning-based autonomous execution. All datasets and evaluations reported in this study were collected on physical hardware; no simulation environments, domain randomization, or synthetic pretraining were used.

The study includes: (i) hardware and sensing design for a biocell manipulation workspace, (ii) a ROS 2-based real-time communication and data collection framework, (iii) an object-query-based perception and conditional diffusion policy for action chunk generation, and (iv) real-world evaluation on the same platform with baseline comparisons.

### 3.2 Experimental platform and biocell environment

#### 3.2.1 Biocell workspace

All experiments were conducted inside a controlled enclosure that emulates indoor agricultural interaction conditions while maintaining repeatability across trials. A tomato plant was placed within the enclosure to introduce realistic biological variability, including foliage-induced occlusions and deformable structures. The enclosure also supports consistent camera mounting and controlled lighting conditions when required.

#### 3.2.2 Robotic manipulators

The platform uses two MyCobot robotic arms, each providing 6 DOF. The arms are arranged in a shared workspace with overlapping reachable volumes to enable coordinated manipulation. The primary operational roles are:

- Actor robot: teleoperated by a human operator to perform task demonstrations.
- Imitator robot: receives commands in real time during teleoperation (for mirroring) and later executes learned policies during autonomous evaluation.

Both robots are controlled in joint position space at a fixed control frequency of 20 Hz, which is sufficient for smooth manipulation while maintaining stable communication and sensor synchronization. Joint limits and safety constraints were enforced throughout data collection and evaluation to prevent collisions and hardware damage.

### 3.3 Teleoperation, communication, and ROS 2 node architecture

**FIGURE 4:**
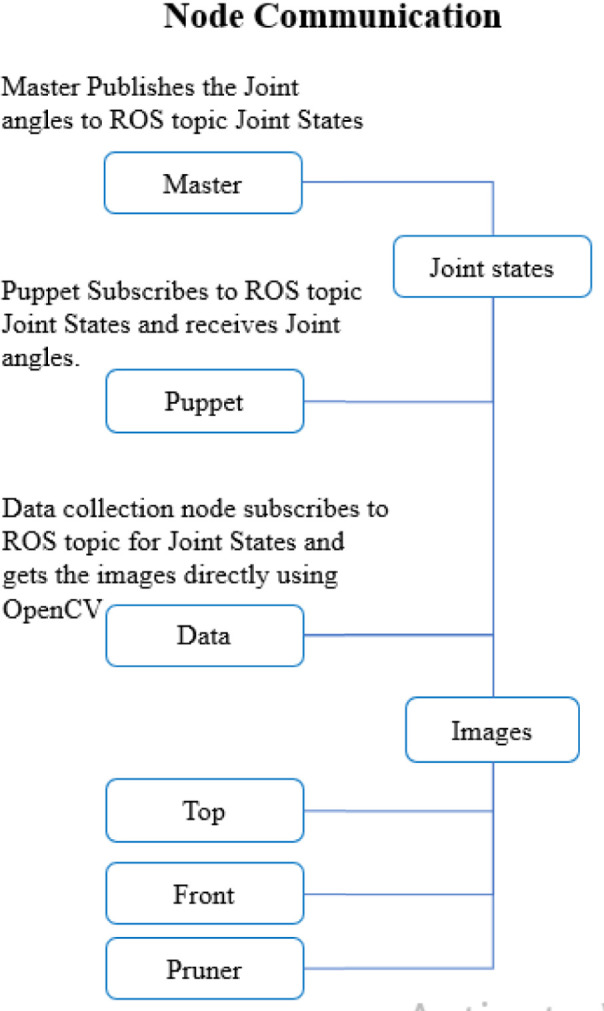
ROS 2-based node communication. The actor robot publishes joint states; the imitator subscribes and mirrors motion. A data collection node records synchronized joint states and multi-camera images with aligned timestamps.

The actor robot is teleoperated by a human operator from a control room using a dedicated interface that converts operator inputs into joint-space commands. Motion execution relies on mirroring and joint-space control strategies to maintain feasible robot configurations during task execution. A WiFi-based communication protocol enables synchronized operation between the actor and imitator robots with low latency, ensuring reliable and temporally consistent transmission of joint commands. The system is implemented using ROS 2 Humble and follows a publisher–subscriber architecture in which the actor robot publishes joint angle data to the ROS topic/joint_states, while the imitator robot subscribes to this topic, parses the received commands, and mirrors the actor’s motion in real time. In parallel, a dedicated data collection node subscribes to the same/joint_states topic while simultaneously recording multi-camera visual observations using OpenCV (Figure 4). All data streams are time-stamped and synchronized to produce aligned demonstrations, supporting both real-time teleoperation monitoring and high-quality dataset generation for subsequent autonomous learning.

### 3.4 Sensors and perception hardware

The imitator robot is equipped with potentiometers for sensing joint angles and positions, providing feedback necessary for accurate motion replication and helping reduce error accumulation during teleoperation and execution. These joint measurements are recorded alongside control commands to maintain a consistent state representation suitable for imitation learning. In addition to joint sensing, the biocell is instrumented with three strategically positioned RGB cameras that provide multi-view observations of the workspace. The cameras are arranged to minimize occlusions and capture the scene from complementary viewpoints, enabling both effective operator monitoring and the collection of visual inputs for learning-based policies. Prior to data collection, camera calibration is performed to ensure consistent geometric alignment between viewpoints, improving cross-view consistency and reliability for downstream perception and learning modules.

### 3.5 End-effector and tooling

For pruning-related agricultural actions, the system employs a custom end-effector specifically designed to perform precise cutting operations. The pruner mechanism uses a pinion-and-rack gear arrangement to convert rotational motion into linear motion, enabling controlled and repeatable cutting trajectories during manipulation tasks. The design prioritizes compactness, mechanical simplicity, and robustness to withstand repeated experimental trials while maintaining reliable performance. This modular configuration also allows potential adaptation of the end-effector for different crop types and agricultural manipulation scenarios.

### 3.6 Task definition

The target task is defined as a coordinated interaction with a tomato plant within the biocell environment. The manipulation sequence begins with the robot localizing the target within cluttered foliage, followed by a synchronized robotic approach toward the plant. Once positioned, the system must achieve a stable grasp for plant handling or engage the pruning tool when cutting actions are required. This is followed by controlled repositioning or interaction with the plant structure. The sequence concludes with a safe disengagement phase that prioritizes collision avoidance and minimizes excessive contact forces.

### 3.7 Dataset collection and synchronization

Demonstrations are captured via an actor–imitator configuration, where the actor robot is teleoperated to perform successful task executions. Throughout each trial, all sensory and control signals are recorded to form a comprehensive dataset. Specifically, each demonstration D is represented as a time-indexed sequence of multimodal observations and control signals. We define the demonstration dataset as follows:Let $I_{t}$ denote the multi-view RGB image observations at time t , and let $s_{t}$ represent the concatenated proprioceptive state, including joint positions, velocities, and end-effector poses for both manipulators. The executed control signal is represented by the action vector $a_{t}$ , which consists of the joint-space commands for both arms. A single demonstration of length T is thus denoted as:

$$D={\left\{\left(I_{t},s_{t},a_{t}\right)\right\}}_{t=1}^{T}$$

Where the constituent vectors are defined as: Visual Observations: $I_{t}=\left\{I_{t,\text{front}},I_{t,\text{left}},I_{t,\text{right}}\right\}$ Proprioceptive State: $s_{t}=\left[q_{t},{\dot{q}}_{t},x_{t}\right]$ (where q , $\dot{q}$ , and x represent joint positions, velocities, and end-effector poses, respectively). Action Vector: $a_{t}=\left[\Delta q_{t,\text{arm}1},\Delta q_{t,\text{arm}2}\right]$ All modalities are temporally aligned via synchronized timestamps to ensure consistency during the imitation learning process.

### 3.8 Object-query-based diffusion policy formulation

To achieve robust robotic manipulation in cluttered environments, we utilize a conditional diffusion policy that integrates object-query-based perception with action chunking. The perception module leverages a DINO-based transformer to extract K object-query embeddings corresponding to detected regions from the input image $I_{t}$ :

$$O_{t}=f_{\text{DINO}}\left(I_{t}\right)=\left\{o_{t}^{1},o_{t}^{2},\ldots ,o_{t}^{K}\right\},\;o_{t}^{k}\in R^{d}$$ (1)

where K denotes the fixed number of object queries produced by the detector. In our implementation, we set K=300 , corresponding to the default number of object queries used in the DINO detection transformer. This fixed query budget ensures consistent representation capacity across all experiments.

Unlike standard patch-based encoders, this formulation yields embeddings corresponding to physical entities in the workspace, providing superior spatial grounding and structural encoding—qualities particularly advantageous for navigating dense foliage. The visual encoder is initialized using pretrained weights, as the use of pretrained encoders is standard in robotics given limited training data. In this setting, the learned representations are effectively adapted through the downstream policy training process. We do not perform a separate, explicit fine-tuning of the encoder, thereby maintaining consistency across experiments and isolating the contribution of the policy architecture. These object tokens are then fused with the concatenated proprioceptive state $s_{t}$ of both arms through a transformer-based fusion module, $f_{\text{cond}}$ , which employs cross-attention to generate a compact multimodal conditioning vector:

$$c_{t}=f_{\text{cond}}\left(O_{t},s_{t}\right)$$ (2)

To mitigate compounding errors and enhance temporal consistency, the policy models a distribution over a sequence of H future actions, defined as an action chunk. To account for non-Markovian dynamics such as plant recoil, $s_{t}$ incorporates a historical window of past states, ensuring that $c_{t}$ captures the temporal context of deformable interactions.

$$a_{t:t+H-1}=\left(a_{t},a_{t+1},\ldots ,a_{t+H-1}\right)$$ (3)

We learn this distribution through a diffusion process. During training, a forward process progressively adds Gaussian noise ϵ to the ground-truth action chunk $a^{0}$ according to a predefined noise schedule $\left\{{\bar{\alpha }}_{\tau }\right\}$ :

$$a_{\tau }=\sqrt{{\bar{\alpha }}_{\tau }}a^{0}+\sqrt{1-{\bar{\alpha }}_{\tau }}\;\epsilon ,\;\epsilon ∼N\left(0,I\right)$$ (4)

A denoising network $\epsilon_{\theta }$ is trained to predict the added noise by minimizing the mean squared error objective:

$$L=E_{a^{0},\epsilon ,\tau }\left[\|\epsilon -\epsilon_{\theta }\left(a_{\tau },c_{t},\tau \right){\|}^{2}\right]$$ (5)

At inference time, the system operates in a receding-horizon manner. The policy generates a denoised action chunk by iteratively sampling from the learned reverse diffusion process conditioned on the current context $c_{t}$ . The first action $a_{t}$ of the generated sequence is executed, and the policy replans at the subsequent timestep, enabling closed-loop feedback and responsive control during manipulation.

### 3.9 Baselines

The proposed method is compared against the following learned policies, trained and evaluated on the same real-world dataset and under identical execution settings:

- CNN-MLP: a convolutional visual encoder followed by a multilayer perceptron action head for joint-space control.
- ViT policy: a patch-based transformer encoder with an MLP action head for action chunk prediction.
- DETR + CVAE: an object-query-based detector combined with a conditional variational autoencoder decoder for action chunk generation.

### 3.10 Implementation and training details

All models are trained using the Adam optimizer for a fixed 4000 epochs, with matched learning rates and batch sizes to ensure performance differences reflect model design rather than optimization. The DINO-based encoder is initialized with pretrained weights, while the task-specific policy layers are trained from scratch. Baseline encoders are similarly initialized using standard pretraining to maintain evaluation fairness. Diffusion-based policies use a linear noise schedule over T diffusion steps, progressively corrupting action sequences with Gaussian noise. This enables the denoising network to recover temporally coherent action chunks, supporting robust multimodal generation and reducing discontinuities or unstable coordination. Training all models for the same number of epochs without early stopping allows direct performance comparison across deterministic, latent-variable, and diffusion-based policies. Conditioning on object-query-based embeddings further leverages structured perceptual information, linking visual features to temporally extended action sequences. This combination of consistent optimization, progressive denoising, and perceptual grounding is crucial for stable execution and robust generalization in cluttered, contact-rich bio-cell environments. We used a fixed 4000 epochs to ensure a consistent basis for comparing convergence behavior across architectures. Importantly, final evaluation results are reported using the best-performing checkpoint for each model selected based on validation performance, rather than the final training epoch.

#### 3.10.1 Encoder initialization and fairness

The detector-based encoder (DINO, based on denoising DETR) is initialized from pretrained weights, consistent with standard practice for object detection models. To ensure a fair comparison, all baseline models are trained using the same dataset, input modalities, and preprocessing pipeline. The visual encoders in baseline models are trained from scratch unless otherwise specified, and no additional external data is used for downstream policy learning. The detector-based encoder is kept fixed during policy training, ensuring that performance improvements arise from the integration of structured object-query representations with the policy architecture rather than from additional representation learning.

#### 3.10.2 Training hyperparameters

All models are trained using the Adam optimizer with a learning rate of $1\times 10^{-5}$ and a batch size of 16. Training is performed for 4000 epochs, and the best-performing checkpoint is selected based on validation performance. The policy network uses a hidden dimension of 512 and a feedforward dimension of 3200. The KL regularization weight of 10 is used for latent-variable baselines.

## 4 Results

This section reports the real-world performance of the proposed object-query-based conditional diffusion policy (DINO + CDP) on a robotic biocell manipulation task using two MyCobot manipulators. While the training data includes robotic-arm trajectories, all evaluations reported here were obtained from single-arm execution by the imitator robot to ensure no skill entanglement occurred during task execution. All evaluations were performed exclusively on physical hardware with identical control frequency, action-chunk horizon, and camera configuration across all methods. The proposed method is compared against three baselines trained and evaluated under the same conditions: a CNN-MLP visuomotor policy, a Vision Transformer (ViT) policy, and a DETR + CVAE action-chunking policy.

### 4.1 Evaluation protocol

Each policy was evaluated over 50 independent real-world trials per experimental condition. Trials were considered independent when the scene configuration was reset, the robotic system was re-initialized, and the task execution was restarted without carrying over prior system states. A trial was classified as successful if the robot completed the target manipulation sequence while maintaining safe operation, defined as the absence of collisions, joint-limit violations, or manual operator intervention. Policies generated action chunks in a receding-horizon manner, where a sequence of future actions was predicted at each timestep and only the first action was executed before replanning.

For statistical analysis, success-rate comparisons between methods were evaluated using Fisher’s exact test due to the categorical nature of the outcome variable and the relatively limited number of trials per condition. Continuous metrics, including execution time, trajectory smoothness, and synchronization error, were analyzed using the Mann–Whitney U test because several distributions deviated from normality under real-world operating conditions. Statistical significance was evaluated at a threshold of p<0.05 . In addition to significance testing, effect sizes and confidence intervals were computed for the primary comparisons to quantify the magnitude and reliability of observed differences between methods.

### 4.2 Metrics

We report task-level and coordination-level metrics. Task success rate is computed as the fraction of successful trials. Execution time is measured from trial start to termination (success or failure). Trajectory smoothness is quantified by mean squared jerk across the concatenated joint positions of both arms:

$$\text{Jerk}=\frac{1}{T-2}\overset{T}{\underset{t=3}{\sum }}{\left\|q_{t}-3q_{t-1}+3q_{t-2}-q_{t-3}\right\|}_{2}^{2}$$ (6)

where $q_{t}$ denotes joint positions and T is the number of executed steps. robotic coordination is measured by synchronization error:

$$E_{\text{sync}}=\frac{1}{T}\overset{T}{\underset{t=1}{\sum }}{\left\|\Delta x_{t}-\Delta x_{t}^{\text{demo}}\right\|}_{2}$$ (7)

where $\Delta x_{t}$ is the relative end-effector pose between arms and $\Delta x_{t}^{\text{demo}}$ is a reference relationship estimated from demonstrations. Safety violations are recorded at the event level, meaning independent occurrences within a single trial are counted separately. These are categorized as: (i) unintended arm-plant contact with non-target regions, and (ii) manual interventions where an operator halts the system due to unsafe behavior. Minor incidental contacts below predefined force/displacement thresholds that do not impede the task are excluded to ensure a transparent measure of performance. While $E_{\text{sync}}$ focuses on pose alignment rather than fully adaptive coordination, it serves as a reliable proxy for task success, as higher alignment consistently correlates with precise tool placement and reduced plant damage.

### 4.3 Training dynamics and generalization gap

**FIGURE 5:**
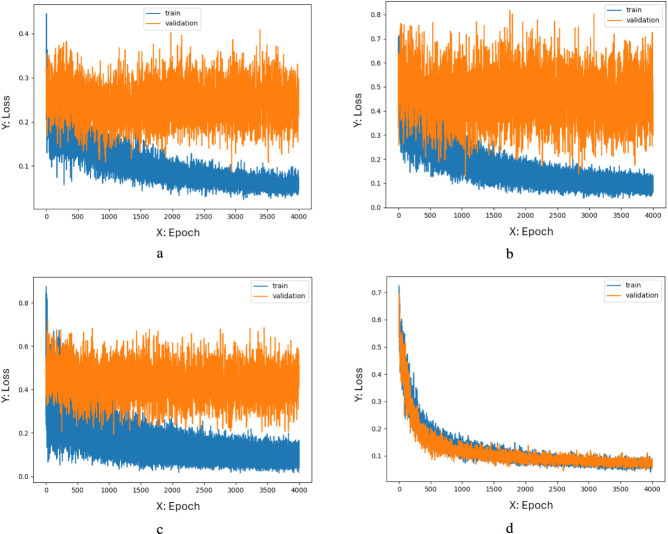
Representative training and validation loss curves across evaluated policies. Baselines often show decreasing training loss with noisy, elevated validation loss, consistent with a generalization gap in real-world trials. The proposed DINO + CDP model exhibits reduced generalization gap, with validation loss decreasing alongside training loss. Panels correspond to CNN-MLP **(a)**, ViT **(b)**, DETR + CVAE **(c)**, and DINO + CDP (**d**:ours). X-axis: Epoch, Y-axis: Loss.

Figure 5 shows representative training and validation loss trajectories for the evaluated models. Several baselines exhibit steadily decreasing training loss while validation loss remains comparatively high and noisy, indicating a pronounced generalization gap under real-world variability (including occlusion from foliage, illumination changes, and small viewpoint or target shifts). In contrast, the proposed DINO + CDP framework shows improved validation behavior, with training and validation curves decreasing together and converging over the course of training.

Because generative action models can exhibit weak correlation between loss values and closed-loop rollout performance, all performance claims are supported by real-robot evaluations using success rate and coordination metrics reported below.

### 4.4 Main quantitative comparison under nominal conditions

Table 2 summarizes nominal-condition performance. The proposed DINO + CDP framework achieves the highest task success rate and improves motion quality and coordination stability relative to all baselines. These improvements are consistent with two complementary mechanisms: (i) object-query-based scene grounding from detection tokens and spatial localization, which reduces perceptual ambiguity in cluttered biological scenes; and (ii) diffusion-based chunk generation that iteratively refines action sequences rather than relying on single-shot decoding, thereby reducing long-horizon drift in coordination.

**TABLE 2:** Performance summary under nominal real-world conditions (mean ± std over 50 independent trials). Higher is better for success; lower is better for execution time, jerk, synchronization error, and safety violations.

Method	Success (%)	Time (s)	Jerk $\left(\times 10^{-2}\right)$	$E_{\text{sync}}$ (cm)	Violations/50
CNN-MLP	62.4 ± 6.1	34.8 ± 7.9	3.20 ± 0.61	4.6 ± 1.2	9
ViT	71.2 ± 5.4	31.1 ± 6.8	2.65 ± 0.55	3.8 ± 1.0	7
DETR + CVAE	79.0 ± 4.7	28.4 ± 6.1	2.10 ± 0.48	3.1 ± 0.9	5
DINO + CDP (ours)	89.1 ± 3.2	24.6 ± 5.4	1.35 ± 0.33	2.0 ± 0.6	2

To further evaluate the statistical significance of the observed performance differences, we conducted pairwise comparisons between the proposed DINO + CDP framework and the evaluated baseline methods. Success-rate comparisons were analyzed using Fisher’s exact test, while continuous metrics were analyzed using the Mann–Whitney U test due to non-Gaussian distributions observed in real-world trials. Table 3 summarizes the corresponding statistical comparisons, including p-values and effect sizes for the primary evaluation metrics.

**TABLE 3:** Statistical comparison between DINO + CDP and baseline methods under nominal real-world conditions. Success-rate comparisons were evaluated using Fisher’s exact test based on 50 independent trials per method. Continuous metrics were analyzed using the Mann–Whitney U test. Statistical significance was defined at p<0.05 .

Comparison	Metric	Statistical test	p-value	Effect size
DINO + CDP vs. CNN-MLP	Success rate	Fisher’s exact	0.002	OR = 5.52
DINO + CDP vs. ViT	Success rate	Fisher’s exact	0.040	OR = 3.50
DINO + CDP vs. DETR + CVAE	Success rate	Fisher’s exact	0.262	OR = 2.25
DINO + CDP vs. CNN-MLP	Trajectory smoothness (Jerk)	Mann–Whitney U	<0.001	r=0.61
DINO + CDP vs. ViT	Trajectory smoothness (Jerk)	Mann–Whitney U	0.006	r=0.48
DINO + CDP vs. DETR + CVAE	Trajectory smoothness (Jerk)	Mann–Whitney U	0.031	r=0.36
DINO + CDP vs. CNN-MLP	Synchronization error	Mann–Whitney U	<0.001	r=0.58
DINO + CDP vs. ViT	Synchronization error	Mann–Whitney U	0.008	r=0.44
DINO + CDP vs. DETR + CVAE	Synchronization error	Mann–Whitney U	0.044	r=0.31

* OR, denotes Odds Ratio.

* r denotes the rank-biserial correlation effect size associated with the Mann–Whitney U test.

The statistical analysis shows that DINO + CDP produced significant improvements over CNN-MLP and ViT in task success rate. Compared with DETR + CVAE, DINO + CDP showed a higher success rate, although the Fisher’s exact test did not reach statistical significance under the 50-trial evaluation. However, DINO + CDP demonstrated significant improvements in trajectory smoothness and synchronization error compared with all baseline methods.

### 4.5 Generalization to real-world variations

To evaluate robustness beyond the nominal configuration, we test controlled real-world variations that occur frequently in the biocell environment. These include target pose shifts (moving the plant target region within a safe workspace range), mild viewpoint perturbations, lighting changes, and increased foliage occlusion. Table 4 reports success rates under these variations. To ensure statistical reliability, all reported success rates are based on multiple independent evaluation trials conducted under consistent conditions, with the mean performance and observed variance reported to account for real-world execution variability. The proposed DINO + CDP model maintains substantially higher robustness than baselines across all conditions, supporting improved transfer under real-world distribution shifts.

**TABLE 4:** Success rate (%) under controlled real-world variations.

Method	Target shift	Viewpoint shift	Lighting shift	Occlusion/Clutter
CNN-MLP	50.1	46.7	53.4	41.2
ViT	60.4	56.1	62.8	52.5
DETR + CVAE	69.2	64.8	71.0	60.6
DINO + CDP (ours)	84.0	81.3	85.2	78.6

### 4.6 Image entropy and scene complexity analysis

To quantify perceptual complexity within the controlled laboratory bio-cell, we compute the Shannon entropy of input RGB images. Unlike open-field agricultural environments, the experimental setup consists of a single plant (or rigid object baseline) placed inside an enclosed chamber with regulated illumination and bounded background surfaces. Consequently, global scene variability is constrained, and entropy primarily arises from object morphology, self-occlusion, texture structure, and induced lighting perturbations.

**FIGURE 6:**
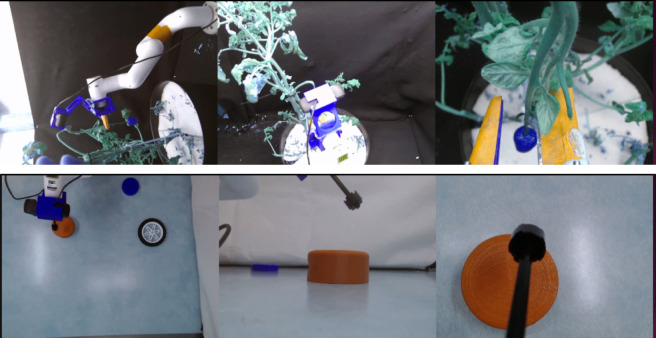
Representative grayscale frames from the bio-cell. Top: Plant scene with overlapping foliage, fine texture structure, and self-occlusion. Bottom: Rigid object scene with smoother surfaces and sharper geometric boundaries.

As illustrated in Figure 6, plant scenes exhibit dense leaf structure, partial translucency, and complex self-occlusions, while rigid-object scenes contain larger homogeneous regions and well-defined edges. These structural differences directly influence grayscale histogram dispersion and thus image entropy.

Image entropy serves as a proxy for pixel-level uncertainty and intensity variability. For each frame, entropy is computed over the grayscale intensity histogram:

$$H\left(I\right)=-\overset{255}{\underset{i=0}{\sum }}p_{i}\log_{2}p_{i}$$ (8)

where $p_{i}$ denotes the probability of intensity level i . Reported values correspond to the mean entropy across frames within a trial, averaged over all trials per condition. While entropy serves here as a comparative proxy for visual complexity, it does not fully capture semantic difficulty; therefore, the true measure of task complexity is evaluated through downstream success rates under varying conditions.

### 4.7 Plant vs. rigid object entropy

To isolate the contribution of organic morphology to scene complexity, we evaluate scenes in which the plant is replaced by rigid, texture-sparse laboratory objects placed in the same enclosure under identical camera and lighting conditions (Figure 6).

Plant-dominant scenes consistently exhibit higher entropy (approximately 0.7–0.9 bits) than rigid-object scenes under identical environmental conditions. This increase is attributable to fine-scale leaf texture, overlapping structures, and complex occlusion patterns, which broaden the grayscale intensity distribution. In contrast, rigid objects produce larger uniform regions and less histogram dispersion.

### 4.8 Policy performance as a function of entropy

To evaluate robustness to visual complexity, plant-scene trials are grouped into entropy bins: low (H<6.6) , medium (6.6≤H<7.0) , and high (H≥7.0) . Success rates are computed separately for each policy.

All policies exhibit performance degradation as entropy increases, indicating sensitivity to pixel-level uncertainty introduced by foliage structure and illumination variability. However, the magnitude of degradation differs substantially across architectures. The CNN-MLP policy shows the steepest decline, suggesting strong sensitivity to local texture statistics. The ViT policy demonstrates improved but still notable degradation. DETR + CVAE exhibits greater robustness, benefiting from object-query structure but remaining affected under high occlusion.

The proposed DINO + CDP framework shows the smallest performance drop (11 percentage points from low to high entropy), indicating reduced sensitivity to perceptual complexity. This suggests that object-query-based representations derived from self-supervised pretraining, combined with conditional diffusion-based action generation, mitigate ambiguity arising from high-entropy visual inputs.

Overall, entropy analysis reveals that even within a controlled indoor bio-cell environment, organic plant morphology induces measurable increases in pixel-level uncertainty relative to rigid objects. Performance degradation scales with entropy across all policies, but the proposed object-grounded diffusion framework demonstrates significantly improved robustness to perceptual complexity (Table 5).

**TABLE 5:** Mean image entropy (bits) in the bio-cell for plant and rigid-object scenes (mean ± std over trials).

Condition	Plant scene	Rigid object scene
Nominal	6.48 ± 0.12	5.72 ± 0.10
Viewpoint shift	6.71 ± 0.18	5.89 ± 0.13
Lighting shift	7.05 ± 0.22	6.21 ± 0.17
Occlusion/clutter	7.28 ± 0.25	6.44 ± 0.19

### 4.9 Inference latency and real-time feasibility

Diffusion policies incur additional compute due to iterative denoising. Table 6 summarizes inference latency measured on the deployment system, decomposed into perception time, policy time, and total time per replanning step. Although diffusion increases policy compute relative to deterministic baselines, the method remains feasible under the receding-horizon control scheme used in our real-robot experiments (Laskey et al., 2017).

**TABLE 6:** Inference latency (ms) measured on the deployment system (per replanning step).

Method	Perception (ms)	Policy (ms)	Total (ms)
CNN-MLP	12.0	2.1	14.1
ViT	18.5	3.2	21.7
DETR + CVAE	26.0	4.6	30.6
DINO + CDP (ours)	28.4	18.1	46.5

### 4.10 Qualitative rollouts and failure modes

Qualitative rollouts corroborate the quantitative trends. Baseline failures frequently arise from incorrect target grounding under foliage occlusion, unstable timing during approach and contact, and oscillatory corrective actions after minor errors. In contrast, DINO + CDP produces smoother trajectories and more stable inter-arm synchronization, particularly during contact-rich phases where coordination errors can compound rapidly. For transparency, we categorize failures into perception-induced errors, interaction/grasp failures, coordination failures, and safety terminations and report representative examples alongside detector overlays and end-effector trajectories in supplementary qualitative figures.

## 5 Discussion

This work investigated whether combining object-query-based perception with diffusion-based action generation improves agricultural manipulation tasks with robotic imitation learning under biological scene variability. The proposed DINO + CDP framework was evaluated exclusively on physical hardware in a biocell environment using two MyCobot manipulators interacting with a tomato plant, and was compared against CNN-MLP, ViT, and DETR + CVAE baselines under matched conditions. The results indicate that DINO + CDP improves task success and produces smoother, more coordinated robotic behavior, while maintaining practical real-time feasibility under a receding-horizon control scheme (Table 7).

**TABLE 7:** Success rate (%) as a function of entropy level (plant scenes).

Method	Low	Medium	High	Drop (Low → High)
CNN-MLP	85	70	48	−37
ViT	88	76	60	−28
DETR + CVAE	90	82	71	−19
DINO + CDP (ours)	92	87	81	−11

### 5.1 Detector-based features improve real-world robotic control

A common failure mode in real-world visuomotor imitation is inaccurate perceptual grounding in cluttered or occluded scenes. CNN-MLP policies rely on global feature pooling, which can blur distinctions between manipulation targets and background clutter. ViT-based policies provide strong representational capacity through attention mechanisms; however, in the absence of explicit object-centric supervision or decoding mechanisms, their patch tokens may not directly correspond to semantically meaningful scene entities. In the biocell environment, the tomato plant introduces occlusion, deformability, and textured variation, making pixel-centric representations prone to brittle attention and unstable manipulation behavior.

The proposed approach instead uses a detector-centric representation that produces object-level embeddings and spatial hypotheses. These object tokens provide a compact interface between perception and control while enabling localized spatial grounding around task-relevant regions. As a result, the policy becomes more robust to visual variation and occlusion, reducing perceptual ambiguity and improving generalization during real-world operation. Furthermore, while this work focuses on visual query stability, the concept of structural robustness—often analyzed through graph-based connectivity (Nethala et al., 2026)—is conceptually related to how consistent object-query representations influence downstream policy behavior. Although a formal graph-theoretic treatment is beyond the scope of this work, such perspectives offer a promising direction for analyzing the reliability of structured perception–control systems in complex biological environments.

### 5.2 Diffusion improves multimodal chunk generation and coordination stability

robotic manipulation admits multiple valid action sequences, and real-world demonstrations exhibit variability in contact timing, grasp approach, and coordination. Deterministic regressors average over this variability, often producing actions that are locally plausible but globally inconsistent, especially for action chunks. Latent-variable models like CVAEs capture multimodality via stochastic latent codes but can suffer from posterior collapse, mode averaging, or instability over long horizons.

Diffusion-based policies instead generate action sequences through iterative denoising of a noisy trajectory conditioned on the observation context. This process reduces discontinuities and enforces globally consistent sequences, which is crucial for coordination where small timing errors can cascade into collisions or failed contacts. The resulting improvements in smoothness and synchronization reflect diffusion’s ability to produce stable, chunk-level action generation with fewer oscillatory corrections than single-shot decoders.

### 5.3 Synergy between object-query-based perception and diffusion policies

The strongest gains in this study are consistent with an interaction effect between structured perception and iterative action generation. Diffusion policies benefit from informative conditioning signals that remain stable under distribution shifts; conversely, object-query embeddings are most valuable when the downstream policy can exploit their structure effectively. In real biological environments, pixel-level noise, occlusions, and minor scene variation can cause large changes in raw feature activations. Conditioning diffusion on stable object tokens can reduce the burden on the denoiser to infer task-relevant structure, improving both training stability and rollout performance. This synergy offers a principled explanation for why DINO + CDP outperforms both pixel-centric baselines and the detector + latent baseline.

### 5.4 Interpretation of generalization results

The generalization results demonstrate that DINO + CDP maintains higher success under target shifts, viewpoint changes, lighting variation, and increased occlusion. These shifts are representative of real-world deployment conditions where the robot must act reliably without curated laboratory uniformity. Robustness to viewpoint and occlusion is particularly important in plant manipulation, where foliage can obscure key target features and where the scene geometry may vary across growth stages. The improved robustness suggests that object-query-based representations provide a more transferable perceptual abstraction than pixel pooling or patch tokenization, and that diffusion-based generation can better accommodate the residual ambiguity that remains even with structured perception.

### 5.5 Practicality and compute trade-offs

A common concern for diffusion-based control is inference latency due to iterative denoising. Our latency measurements indicate that diffusion introduces additional policy compute relative to deterministic baselines. However, the overall approach remains feasible in practice because control is executed in a receding-horizon manner: the system replans periodically, and only the first action in each predicted chunk is executed before generating a new chunk. This design allows the policy to trade off between denoising steps and responsiveness while maintaining stable closed-loop behavior. In real deployments, diffusion steps can be tuned to match control-frequency constraints, and further acceleration techniques (e.g., fewer-step samplers, distillation, or lightweight denoisers) can be applied when necessary.

### 5.6 Limitations

This study has several limitations. First, while the bio-cell setup provides a realistic and challenging environment, it represents a specific task domain; additional tasks involving different plant structures, end-effector tools, or contact dynamics would strengthen claims of broad generality. Additionally, while our dataset captures structural variability in foliage and density, it is currently limited to a single growth stage. The model’s ability to generalize across the full developmental lifecycle—where stem rigidity, branching patterns, and plant morphology evolve significantly—has not yet been formally established. Second, the dataset size and diversity of demonstrations affect the degree of multimodality the policy must model; future work should evaluate scaling trends as the number of demonstrations increases. Third, diffusion policies can be sensitive to hyperparameters such as noise schedules, denoising steps, and chunk horizons; although the presented framework demonstrates strong performance, systematic hyperparameter studies are necessary for robust deployment guidelines. Finally, detector-based perception can fail under severe occlusion or extreme lighting, and future work should incorporate temporal tracking or multi-view fusion strategies that explicitly handle missed detections.

### 5.7 Future work

Several extensions are promising. First, expanding to additional manipulation primitives and plant varieties would evaluate whether the learned object-query-based abstractions generalize across diverse biological structures, crop geometries, and growth stages. Second, integrating explicit temporal tracking of object tokens across frames may improve stability under transient occlusion, leaf motion, and partial viewpoint changes common in agricultural environments. Third, extending the framework to larger robotic arm systems (beyond two manipulators) would test the scalability of object-query-based diffusion conditioning in higher-dimensional coordination settings, particularly for cooperative manipulation tasks.

A key future direction involves deploying the proposed system on a mobile manipulation platform capable of navigating within open fields or greenhouse environments. Unlike the current fixed biocell setup, a mobile version would introduce additional challenges including dynamic viewpoint changes, uneven terrain, navigation–manipulation coupling, and larger environmental variability. Integrating autonomous navigation with object-query-based perception would require joint optimization of localization, mapping, and manipulation policies, potentially leveraging diffusion models conditioned on both scene-level and workspace-level context. Such a system would enable continuous operation across multiple plants and rows, significantly increasing throughput while preserving precise coordination.

Furthermore, combining diffusion-based imitation with online adaptation or low-risk reinforcement learning could improve robustness to previously unseen conditions while preserving safety constraints. Finally, developing accelerated diffusion samplers tailored to real-time robotics—such as reduced-step denoising or learned deterministic approximations—could further reduce inference latency while maintaining multimodal action generation capability, which becomes particularly important for mobile manipulation scenarios where perception and control must operate under tighter timing constraints.

Overall, the results support the conclusion that object-query-based perception combined with diffusion-based action generation provides a robust and scalable approach for real-world robotic imitation learning in biologically complex environments and offers a strong foundation for future deployment in mobile and large-scale agricultural robotic systems.

## Data Availability

The datasets presented in this study will be provided upon reasonable request.
